# CRISPR/Cas9 system in breast cancer therapy: advancement, limitations and future scope

**DOI:** 10.1186/s12935-022-02654-3

**Published:** 2022-07-25

**Authors:** Vamika Karn, Sandhya Sandhya, Wayne Hsu, Deepak Parashar, Himanshu Narayan Singh, Niraj Kumar Jha, Saurabh Gupta, Navneet Kumar Dubey, Sanjay Kumar

**Affiliations:** 1 Department of Biotechnology, Amity University, Mumbai, 410221 India; 2 Division of Oncology Research, Mayo Clinic, Rochester, MN 55905 USA; 3 Division of General Surgery, Department of Surgery, Taipei Medical University Hospital, Taipei, 110 Taiwan; 4 Department of Obstetrics and Gynaecology, Medical College of Wisconsin, Milwaukee, WI 53226 USA; 5 Department of System Biology, Columbia University Irving Medical Centre, New York, NY 10032 USA; 6 Department of Biotechnology, School of Engineering & Technology (SET), Sharda University, Greater Noida, 201310 India; 7 Department of Biotechnology, School of Applied & Life Sciences (SALS), Uttaranchal University, Dehradun, 248007 India; 8 Department of Biotechnology Engineering and Food Technology, Chandigarh University, Mohali, 140413 India; 9 Department of Biotechnology, GLA University, Mathura, Uttar Pradesh India; 10 Victory Biotechnology Co., Ltd., Taipei, 114757 Taiwan; 11 ShiNeo Technology Co., Ltd., New Taipei City, 24262 Taiwan; 12 Department of Life Sciences, School of Basic Sciences and Research, Sharda University, Greater Noida, 201310 India

**Keywords:** CRISPR/Cas9, Breast cancer, Gene editing, Immunotherapy, Diagnosis, Drug resistance

## Abstract

Cancer is one of the major causes of mortality worldwide, therefore it is considered a major health concern. Breast cancer is the most frequent type of cancer which affects women on a global scale. Various current treatment strategies have been implicated for breast cancer therapy that includes surgical removal, radiation therapy, hormonal therapy, chemotherapy, and targeted biological therapy. However, constant effort is being made to introduce novel therapies with minimal toxicity. Gene therapy is one of the promising tools, to rectify defective genes and cure various cancers. In recent years, a novel genome engineering technology, namely the clustered regularly interspaced short palindromic repeat (CRISPR)-associated protein-9 (Cas9) has emerged as a gene-editing tool and transformed genome-editing techniques in a wide range of biological domains including human cancer research and gene therapy. This could be attributed to its versatile characteristics such as high specificity, precision, time-saving and cost-effective methodologies with minimal risk. In the present review, we highlight the role of CRISPR/Cas9 as a targeted therapy to tackle drug resistance, improve immunotherapy for breast cancer.

## Introduction

Breast cancer (BC) is the most common cause of cancer-related death and also a second leading etiology of mortality among women [1, 2]. Apart from its higher occurrence in females with approximately 28% of new cancers, relatively rare cases in males have also been reported [3]. BC is a disorder with a high degree of heterogeneity and dysregulated signalling pathways, which may drive its onset and development [4]. Clinical heterogeneityis caused by hereditary and somatic changes by 10% and 90% of BC cases, respectively [5]. The prognosis of metastatic BC is still a challenging task at the histopathological and molecular level [5]. BC metastasis is influenced by abnormal gene expression, which results in the activation of downstream signal pathways [6]. Germline mutations are more common in genes irrespective of their penetrance sensitivity [7], while somatic variations are developed during life and include both genomic mutations and epigenetic dysregulation [8]. These changes at the genetic and epigenetic levels orchestrate the cancer cell metabolic requirements such as altered lipid metabolism, leading to enhanced cancer cell proliferation and tumorigenesis [9]. However, CRISPR/Cas9 is becoming a promising therapeutic tool, and its diverse applications make it vital even in BC research [10]. The Cas9 nuclease associated with CRISPR allows for the precise insertion, correction, or removal of defective genes. There are some existing limitations also associated with this technique for all types of cancer; nonetheless, progress is being made to overcome them. Taken together, the current review highlights the various therapeutic approaches in BC through CRISPR/Cas9.

## CRISPR/Cas9: the background

**Fig. 1:**
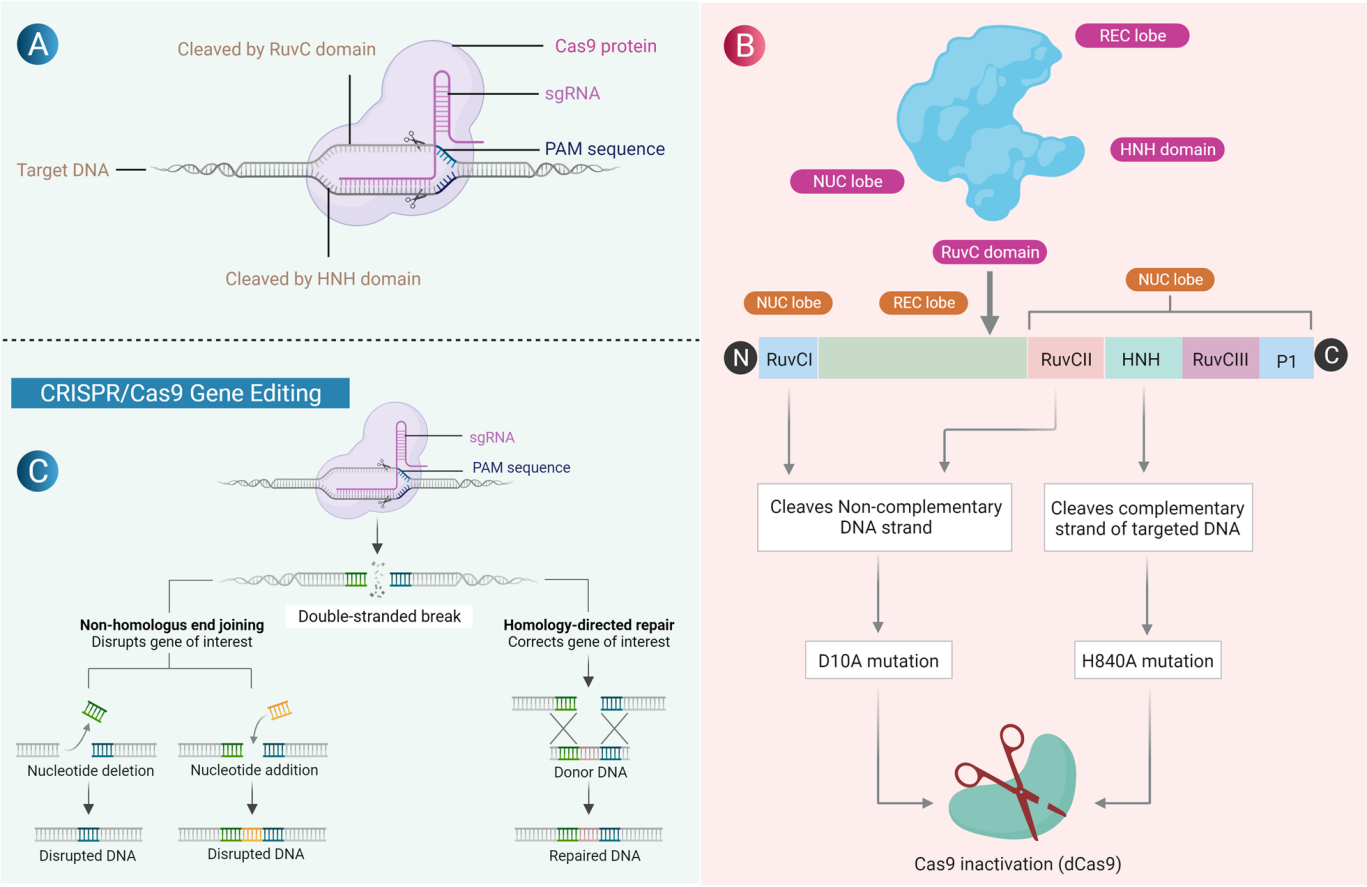
**A** Components of the CRISPR/Cas9 system: (i) Cas9 endonuclease which is responsible for cleavage of target DNA sequence, (ii) single guide (sg) RNA formed by the fusion of crRNA and tra-crRNA chimera, iii) protospacer adjacent motif (PAM) sequence required for Cas binding present in the target DNA sequence. **B** Cas9 protein is a bi-lobed structure consisting of the alpha lobe and the nuclease lobe. The nuclease lobe has two domains the HNH domain and RuvC domain which cleaves the complementary and the non-complementary strands of DNA respectively. Mutation at D10A in the RuvC domain and H840A of the HNH domain leads to the inactivation of Cas9 (dCas9). **C** Gene editing; Cas9-sgRNA complex targets the respective gene and causes double-strand breaks (DSBs) close to the PAM region. The damaged DNA is repaired either by non-homologous end joining (NHEJ) or the homologous DNA repair (HDR) pathway

CRISPR/Cas system is a phenomenal gene-editing tool and referred as genetic scissors. Its potential in precise editing of the DNA revolutionized basic science research. All CRISPR/Cas systems have three principal components: (i) a guide RNA or CrRNA is a unique non-coding RNA, which directs the CRISPR/Cas complex to the target DNA, (ii) auxiliary trans-activating crRNA named as tracrRNA. CrRNA and tracrRNA fused to form chimera is termed as single-guide RNA (sgRNA), (iii) The Cas protein, an endonuclease that mediates the tailoring of target DNA sequences [10]. Further, Cas nuclease needs a specific sequence, known as a protospacer adjacent motif (PAM) to cleave the target DNA sequence (Fig. 1A).

To precisely manipulate the DNA sequences, sgRNA plays a fundamental role in ensuring the editing at the desired locus in the target. The sgRNA is composed of two components known as scaffold sequence and ∼ 20 nucleotide spacers. Scaffold sequence is very important for Cas protein binding, and spacer sequence shares the homology with the target sequence. The seed sequence in the spacer is the first 10–12 nucleotides of sgRNA at the 3′-end close to a PAM sequence that directs the Cas9 nuclease to the target sequence in the genome. The mismatch in seed sequence aborts the interaction between CRISPR/Cas and target DNA sequence, therefore abolishing the DNA editing. Hence, sgRNA defines the target and plays a crucial role in the specificity, efficiency, and precision of the CRISPR/Cas-mediated gene manipulations [11]. The optimal nucleotide length is required for the specificity and efficiency of sgRNA. Recent studies also revealed that a sgRNA with fewer than 20 nucleotides significantly reduced nonspecific DNA editing by maintaining its efficiency [12]. Furthermore, another study found that the formation of around 5 base pairs in the sgRNA duplex can significantly improve CRISPR/Cas9 knockout proficiency [13].

Structurally, the Cas9 peptide contains the recognition (REC) and nuclease (NUC) lobes [14]. The REC lobe is vital for sgRNA and DNA binding, whereas the NUC lobe is comprised of RuvC and the HNH domains [14]. The HNH and RuvC domains have nuclease activity and nick the complementary and non-complementary target DNA strand, respectively, and create a DNA double-strand break (Fig. 1B). It has been reported that mutations may occur in both catalytic domains (D10A for RuvC and H840A for HNH in S. pyogenes Cas9) and results in the inactivated form of Cas9 known as catalytically dead Cas9 (dCas9), which is not capable of cleaving the target DNA sequence like Cas9 (Fig. 1B). The binding of dCas9/sgRNA to the target DNA sequence blocks the RNA polymerase binding and interferes with the transcription mechanisms [15].

## CRISPR/Cas9 based gene editing for breast cancer therapy

CRISPR/Cas9 has been widely used in basic and translational research in the field of cancer biology. The technique can be used to target oncogenes and tumour suppressor genes (TSG) to reduce cancer progression through various mechanisms. The target can be achieved by knocking out, gene editing, repression, and epigenetic modifications (Table 1). The mechanism of gene editing using the CRISPR/Cas9 tool involves either by non-homologous end-joining (NHEJ) or homology-directed repair (HDR) pathway (Fig. 1C). NHEJ pathway is more frequent in most cell types and involves insertion or deletion of nucleotide bases randomly at the cleavage site in double-strand breaks (DSBs). This is an error-prone DNA repair pathway as it causes frame shift mutations, leading to the synthesis of premature/non-functional polypeptide. In contrast, HDR pathways are error-free and use the homologous region of the donor DNA template to rectify DNA damages [16]. CRISPR/Cas9 has been effectively used for knocking out both cellular as well as viral oncogenes in various cancer models such as leukaemia [17], cervical cancer [18], prostate cancer [19], endometrial cancer [20], ovarian cancer [21] and breast cancer [22].

**Table 1:** Recent studies highlighting different altered gene using CRISPR/Cas9 for BC therapy

Target Gene	Cell line	CRISPR approach	Effects	References
MYC Oncogene	–	CRISPR/Cas9-mediated mutagenesis	Decreased MYC expression and cell proliferation	[23]
CXCR7 and CXCR4	MDA-MB-231	CRISPR/Cas9 knockout	decreased tumor cell proliferation, invasion, and tumor growth	[26]
PTEN	SUM159	CRISPR activation	Lowers cancer aggressiveness	[27]
miRNA23b and miR27b	MCF-7	CRISPR/Cas9 knockout	Decreased tumor growth	[29]
MASTL	Human mammary tumor cell lines	CRISPR-based interruption	Restricts cell proliferation	[37]
FASN	MCF-7	Type 2 CRISPR/Cas9	Inhibits cell proliferation, survival,growth, cell cycle, migration, cell adhesion, and DNA replication	[39]
CDK7	TNBC cell lines	CRISPR/Cas9 genetic editing	Inhibits cell growth and tumorigenesis	[44]

### CRISPR/Cas9 targeting MYC gene

In a study by Schuijers et al. CRISPR/Cas9 to downregulate the MYC gene, which has been reported to have a higher expression with a 30–50% increase in high-grade breast cancers [23]. Therefore, cMYC has been considered as a foremost target in cancer therapy. However, pharmacological inhibition of cMYC is challenging. Usually, super-enhancer genes are bound by MYC. Therefore, the inhibitors of super enhancer might inhibit the cancer cell proliferation, migration and invasion by suppressing the MYC target genes such as CDK6 and TGFβ2 [24]. Additionally, CRISPR/Cas9-mediated deletion of either MYC enhancer-docking site or epigenetic modifications of MYC regulatory elements has shown to impede TF binding and decrease the levels of MYC protein expression. In vitro downregulation of MYC in cancer cells has been associated with reduced cell proliferation [23].

CRISPR interference (CRISPRi) and CRISPR activation (CRISPRa) are other arms of CRISPR/Cas, to repress or activate genes. Specifically, the CRISPRi has been implicated to suppress the oncogenes, whereas CRISPRa activates tumor suppressor genes (TSGs), and both of the techniques can be employed in BC treatment. In CRISPRa, a chimera of dCas9 could also be used, which is the combination of transcriptional activators, and demethylase [25]. The possible ways of TSGs downregulation could be the hypermethylation at promoter site,which lead to the dysregulation of many TSGs-associated TFs. CRISPRa could activate these suppressed genes as well as PTEN in SUM159 cells. PTEN is a TSGs, and its loss has been reported to be more aggressive phenotypes of BC [26]. CRISPR/Cas9 may demethylate the promoter while activating the gene. The dCas9-TET chimera which is a fusion of dCas9 with a Ten-Eleven Translocation (TET) dioxygenase1 (TET1CD) were utilized to demethylate the BRCA1 promoter in vitro, which leads to activation and upregulation of BRCA1 [27]. In other study by Lu A et al., found that, fusion of dCas9 with an R2 stem-loop, a short RNA sequence that selects and impedes the DNA methyl transferase 1 (DNMT1) protein expression, which results in reduced cancer growth by increased demethylation [28].

### CRISPR/Cas9 targeting CXCR7 and CXCR4

Furthermore, Yang et al. used CRISPR/ Cas9 technology to create either CXCR7 or CXCR4 knockout or co-knockout in MDA-MB-231 breast cancer cells [29]. A clinical study reported that the higher expression of CXCL12 and its receptors CXCR4 and CXCR7 has been correlated with higher susceptibility to metastasis and poor prognosis of TNBC [30]. CXCR4 and CXCR7 may stimulate cell motility, invasion, angiogenesis, and tumorigenesis [31]. The findings in Yang et al. study also revealed that co-knock out of CXCR4 and CXCR7 could significantly suppress TNBC, suggesting the synergistic role of CXCR4 and CXCR7 in the advancement of TNBC [29]. CRISPR/Cas9 could not only generate a knockdown protein-coding gene but also edit or delete noncoding RNA regions. Hannafon et al. created CRISPR/Cas9-derived miR-23b and miR-27b miRNA knockout MCF-7 cell line, and they found the oncogenic potential of both of these miRNAs but under certain circumstances, miR-27b may adopt tumour suppressor activity as well [32].

### CRISPR/Cas9 targeting cell cycle kinase MASTL

For the last few years, cell cycle kinase MASTL (also called as Greatwall) has been an emerging player in regulating Protein phosphatase 2A (PP2A) for mitotic division. The altered expression and somatic mutations of PP2A subunit B55 has been identified many cancers including BC [33]. MASTL involves in phosphorylation of two other proteins, endosulfine (ENSA) and ARPP19, which further binds and inhibits PP2A-B55 complexes [34]. Additionally, PP2A-B55 complexes has been also shown to be influenced by CDK substrates phosphorylation [35]. Hence, inhibiting MASTL and reactivating PP2A is very important for maintaining the normal growth of cells by exiting mitotic cell division [36]. Alvares-Fernández et al. has demonstrated the inhibition of MASTL kinase activity using CRISPR/Cas9 could reduce cell proliferation in human cell lines as MASTL was known to overexpress in cancer cells when compared to non-cancerous cells in vivo [37]. Moreover, the inhibition of MASTL using CRISPR/Cas9 could be an important therapeutic approach in the management of BC.

### CRISPR/Cas9 targeting FASN gene

FASN gene encodes fatty acid synthase for the de-novo synthesis of fatty acids, which are primarily needed for the production of phospholipids, and have been considered as an oncogene [9]. Reports have shown a positive correlation for FASN protein expression during the progression of different cancers such as stomach, lung, breast, prostate, colon, and ovarian carcinomas. Increased FASN fulfils the phospholipids requirement for dividing cancer cells and plays a crucial role in cancer cell development and proliferation, hence it may be an appealing target for cancer therapy [38]. Furthermore, the impact of CRISPR/Cas9-based FASN mutants has also been evaluated in MCF-7, a BC cell line. Results demonstrated that mutant FASN exerts an inhibitory effect on MCF-7 progression, implying that FASN mutations have a non-redundant function in BC [39].

### CRISPR/Cas9 targeting HER2 gene

Another oncoprotein HER2 has been delineated using CRISPR and identified as a potential target for cancer therapy. Additionally, HER2 in BC are of great importance as about one-fifth of BC patients have extra copies of the HER2 gene and its overexpression renders it more aggressive in BC than other types of cancers. The mutation in HER2 exon12 has been reported as a dominant-negative mutant phenotype and may suppress the HER2-MAPK/ERK pathway. This effect was potentiated by the treatment of poly-ADP ribose polymerase (PARP) inhibitors [40]. CRISPR/Cas9-mediated editing in one copy of HER2 does not affect overall HER2 protein production, indicating that incomplete HER2 mutations.

### CRISPR/Cas9 targeting FOXA1

Fork-head box protein A (FOXA1) is pioneer transcription factor, that regulates the organogenesis and development of various malignancies including BC. Transcriptional gene regulation is influenced by the binding of transcription factors. FOXA1 binds approximately 90% of the total different genes available in the human cancer genome. However, only around 17% of this FOXA1 involves in transcriptional regulation of genes and these functional FOXA1 are specific to types of cancer cell [41]. FOXA1 marks genomic signatures in a cell-specific manner and regulates gene expression differentially in different human cancer cell lines [42]. This differential regulation could be due to varying epigenetic regulations such as histone modifications. CRISPR/Cas9-mediated editing of cell-specific FOXA1 regulation identifies the unique FOXA1 binding, genetic variations, and potential epigenetic regulation. Additionally, CRISPR technology has been employed in editing FOXA1 binding sites and manipulate the cell specific gene transcription, which leads to reduced BC cancer progression [42]. TNBC is regulated by ubiquitous transcriptional process and a few cyclin-dependent kinases (CDKs) and these CDKs are investigated by CRISPR/Cas genome editing tool [43]. The inhibition of CDK7 can target cancer cells, leading to their apoptosis [44].

### CRISPR/Cas9 targeting CDK7

The cyclin-dependent kinase 7 (CDK7) is the catalytic subunit of CDK-activating kinase (CAK), that involves in catalysis process for phosphorylation of T loops and activates other various cyclin-associated kinases such as CDK1, CDK2, CDK4, and CDK6. CDK7 has been shown to be very crucial for the TNBC development and progression [45]. The CRISPR/Cas9-mediated inactivation of the CDK7 gene in TNBC preferentially impedes cell growth and tumour formation. CRISPR/Cas9 knock-outs of other known CDKs (8, 9, 12, 13, and 19) have been correlated in transcriptional regulation, implying that CDK7 is primarily important for sustaining and proliferation of TNBC cells [44]. Using CRISPR/Cas9-mediated knock-out of TNBC driver genes such as EGFR, FOSL1, FOXC1, MYC, and SOX9 has revealed that CDK7 regulates these TNBC driver genes, which are key in cell proliferation [44]. These shreds of evidence suggest that CRISPR/Cas9 could be used as a therapeutic tool to target CDK7 in controlling cancer cell growth.

### CRISPR/Cas9 targeting UBR5

UBR5 is a nuclear phosphoprotein, has been found upregulated in TNBC samples and a key regulator in the pathway involved in developing resistance for endocrine therapy in TNBC via upregulation of unfolded proteins and reduced degradation of ERα, an important protein for TNC development and progression [46]. Furthermore, CRISPR/Cas9-driven knockout of E3-Ubiquitin Ligase (UBR5) deregulate the tumor growth and metastasis in vivo murine mammary model of TNBC [47]. Hence UBR5 has been considered as a driver for tumor growth and metastasis in BC.

### CRISPR/Cas9 activates tumor suppressor genes

FOXP3 is a X-linked tumor suppressor genes, and heterozygous mutation in FOXP3 has been shown to develop BC in the mice model [48]. In BC, the expression of FOXP3 were reduced, thus by utilizing CRISPRi/a technology, endogenous FOXP3 could be reactivated to upregulate their expression, which lead to reduced BC growth [49]. Also, it has been reported that breast organoids could be developed from normal breast epithelial subsets, which can be altered genetically using CRISPR/Cas9 to form tumors. CRISPR/Cas9 was employed to edit four tumor suppressor (P53, PTEN, RB1, and NF1) genes in breast organoids, resulting into the development of ER-α luminal breast cancer [50], which indicated that the inactivation of these four tumor suppressor genes are key drivers in generating BC under in vivo condition. Thus, CRISPRi/a-driven activation of these tumor suppressor genes may provide a better understanding for its regulatory pathways in designing treatment strategy in the management of BC.

## CRISPR/Cas based immunotherapy

**Fig. 2:**
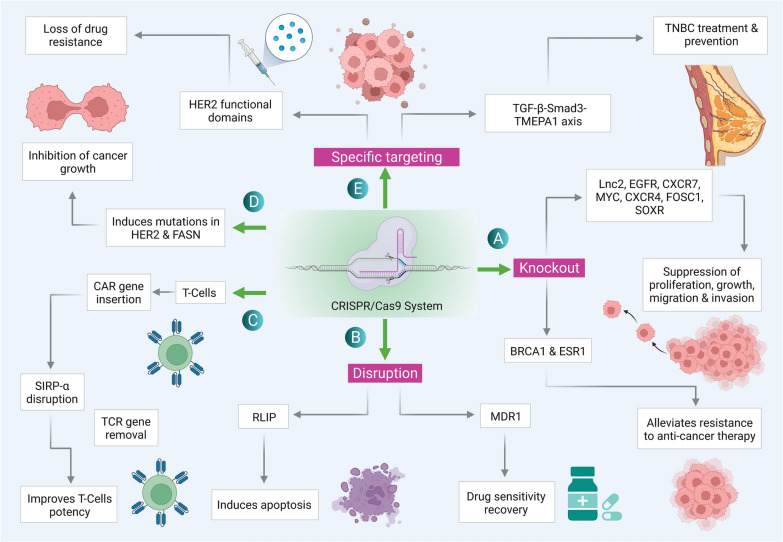
Application of CRISPR/Cas9 system in the treatment of cancer: **A** Knock-out of various oncogenes whose overexpression or dysregulation leads to either resistance to therapy or cancer proliferation. **B** Genes RLIP and MDR1 are responsible for drug resistance in BC are disrupted using CRISPR/Cas for restoration of drug sensitivity. **C** T-cells are used for immunotherapy in BC, CRISPR/Cas has been applied in T-cells for CAR gene insertion, TCR gene removal, and SIRP-α disruption and therefore improving its potency. **D** Mutation in HER2 (human epidermal growth factor receptor 2) and FASN (Fatty acid synthase) induced by CRISPR/Cas9, leads to inhibition of growth of cancer cells. **E** TGF-Smad3-TMEPAI axis plays a role in cancer cells by enabling them to escape TGF-mediated growth inhibition and the functional domains of HER2 are required for carcinogenic activity, hence their specific targeting through CRISPR/Cas results in TNBC treatment and loss of drug resistance respectively

The impaired immune mechanism is a key factor in tumour development. Cancer cells employ various strategies to avoid the defence mechanism through interfering immune cell function and rendering the tumour microenvironment immune-compromised. Therefore, to target the cancerous cells, enhanced immune system has been considered as an important strategy (Table 2). CRISPR/Cas-based genetic alteration has been attempted to resolve a few concerns associated with immune dysfunction for various perspectives [51]. CRISPR/Cas9 has been employed to enhance the anti-tumor immunity against BC via following mechanism (Fig. 2).

**Table 2:** Application of CRISPR in immunotherapy and drug sensitization by targeting different genes

Target Gene	Cell/Cell line/ Animal Model	Effects	References
Replacement of TCR with CAR	T-cells	Improves T cell potency, reduce terminal differentiation, and depletion of lymph nodes	[51, 53]
SIRP-α silencing	Macrophages	Incapable of receiving “do not eat me” signal leading to the destruction of cancer cells	[54]
p38	Mouse models of established tumors	Improve T cell anti-tumor functionalities for ACT	[56]
Cdk5 knockout	TNBC	Downregulated PD-L1 expression Tumor growth inhibition in murine melanoma Lung metastasis suppression in TNBC	[57]
PI3K	–	Overcomes chemo-resistance	[69]
APLNR deletion	Animal models	Reduces the sensitivity and efficacy of checkpoint blockade	[73]
MALAT1 promoter deletion	BT-549 TNBC model	Increases susceptibility to paclitaxel and doxorubicin	[77]
MDR1	MCF-7/ADR cells	Elimination of doxorubicin resistance	[80]
RLIP disruption	–	RLIP downregulation induces apoptosis via both drug-dependent and drug-independent mechanisms	[90]

### CRISPR/Cas 9 in T cell-based immunotherapy

Currently targeting T-lymphocytes to treat cancer is very appealing as this exhibits the propensity to differentiate between self and non-self due to their vast T cell repertoire. Usually, immunotherapy, the patient T cells are engineered to express T cell receptor (TCR) and Chimeric Antigen Receptors T cells (CAR T cells) to recognize Tumour-Associated Antigen (TAA) [52]. This process is time-consuming and mostly depends upon the nature of patients' T-cells [51, 53]. CRISPR/Cas9 has been utilized to replace the TCR with CAR by introducing the CAR gene into the T-cell receptor α constant (TRAC) locus [53]. The ectopic CAR expression in T cells could improve T cell potency, reduce terminal differentiation, and depletion of lymph nodes in a mouse model of AML [51]. Hence this CRISPR/Cas 9 could also utilized to enhance T-cell potency to tumor growth of BC.

### CRISPR/Cas 9 in producing genetically modified T cell

Moreover, T lymphocytes can be genetically modified for expressing cancer antigen-specific T cell receptors (TCR). To rule out the effect of endogenous TCRs in recognizing the cancer cells, the endogenous TCR-β has been knocked out from the recipient cells using CRISPR/Cas9. The CRISPR-edited T cells have demonstrated thousand times more responsiveness towards cancer antigens as compared to the normal TCR-transduced T cells [54]. Similarly, CAR T-cells have been modified with CRISPR/Cas9 to generate inhibition-resistant universal CAR-T cells [55].

### CRISPR/Cas 9 in screening primary T cells

T cells are driving player among available effective immunotherapies for cancers, and their anti-tumor property depend upon their involvement in cell expansion, differentiation, oxidative stress, and genomic stress. Using genetic based CRISPR/Cas9 screening of primary T cells, disrupted 25 T cell receptor-driven kinases were identified. [56]. Of them they found p38 kinase as a central regulator for cell expansion, differentiation, and oxidative stress. The higher p38α levels resulted in rigorous cellular expansion, minimal oxidative, genomic stress, and terminal differentiation. Therefore, the pharmacological inhibition of p38 could enhance the anti-tumour activity of T cells, indicating its potential to be a therapeutic agent for cancer [56].

### CRISPR/Cas 9 in targeting PD-L1

Besides others, monoclonal antibody-based targeting of programmed death-ligand 1 (PD-L1) on tumour cells has been a promising approach for cancer treatment for the last many years. In tumour cells, higher expression of PD-L1 has been reported, and the interaction between programmed cell death 1 receptor (PD1) present on immune cells and its ligand PD-L1 may promote immune evasion and formation of the immunosuppressive tumour microenvironment. The approach employing monoclonal antibodies to block the engagement of PD-L1 with PD-1 in a clinical trial has not been very promising though it provided the future scope to develop cancer therapeutics, which might improve the clinical trial results. Deng et al. have shown that CRISPR-Cas9-assisted downregulation of PD-L1 levels on tumour cells by knocking out the Cdk5 gene might also significantly inhibit murine melanoma growth and suppress lung metastasis in TNBC [57]. Further, a substantial increase in T cell-mediated immune responses in the tumour microenvironment, with increased CD8 + T cells and decreased regulatory T cells population was also noted. Hence, this could be an alternative therapeutic strategy based on immune checkpoint blockade.

### CRISPR/Cas9-mediated alteration of macrophages

Immunological macrophages have been known for various roles in cancers. The interaction of macrophage signal-regulatory protein alpha (SIRP-α) with a glycoprotein CD47 direct signal for “do not eat me”, which helps cancer cells to grow during immune evasion [54]. The induced CD47 expression has been reported in various cancers such as ovarian carcinoma, murine myeloid leukaemia, and leukemic stem cells [58]. Therefore, employing CRISPR/Cas9-mediated alteration of macrophages lacking signal-regulatory protein alpha (SIRP-α) could be a novel strategy to combat BC. This could be achieved via the inability of receiving the “do not eat me” signal by altered macrophages from the cancer cells, leading to their phagocytosis [54] (Fig. 2).

### CRISPR/Cas9 in anti-tumor immunity

Immunotherapy has revolutionized cancer therapy. Although, existing immunotherapies have several limitations. To overcome this, multiplexed activation of endogenous genes as an immunotherapy (MAEGI), was employed as a novel immunotherapeutic approach that induces anti-tumor immunity via CRISPRa (CRISPR activation)-mediated multiplexed activation of endogenous genes. This results into the presentation of tumor antigens, leading to dramatic increase in anti-tumor immune responses. Utilizing this technology as a cell-based vaccination approach, efficacy of prophylaxis and therapeutic property of vaccines could be enhanced. Further, this treatment strategy may also lead to remodelling of the tumor microenvironment and enhanced T cell recruitment. Hence, MAEGI could be a more versatile therapeutic approach in enhancing potent immune responses against breast cancer than current available therapies [59].

## Role of CRISPR/Cas in overcoming chemoresistance

BC is categorized mainly into four subtypes according to various markers such as progesterone receptor (PR), estrogen receptor (ER), ERBB2 (HER2), p53, and Ki-67 [60]. Chemotherapeutic resistance has been evidenced in approximately 30% of BC patients with ER-positive subtypes [61]. Hence, targeting the driver genes involved and understanding of mechanisms for drug resistance are very crucial for clinicians to prescribe the potential drugs for BC therapy. These could be achieved either by knocking down the driver genes or by sensitizing BC cells to chemotherapy. CRISPR/Cas9 is being utilized not only to find new therapeutic targets but also to investigate the causes of drug resistance in cancer chemotherapy (Table 2).

### CRISPR/Cas9 in neutralizing drug resistance factor

CRISPR/Cas9 technology could be one of the choices in mitigating resistance of these factors through multiple ways, such as targeting membrane transporters and enhancing DNA repair and efflux mechanisms [62]. A mouse model, the function of transporter resistance protein has been shown to improve through CRISPR/Cas9 [63]. Likewise, the PTEN-knockout can elevate the expression of ATP-binding cassette G subfamily (ABCG) transporter proteins [64]. The reduced level of PTEN has been associated with enhanced cancer sensitivity to mTOR inhibitors and could be a potential target for CRISPR/Cas9-mediated gene editing in BC [65, 66].Patients with defective BRCA1/2 genes have poor DNA repair capacity and are more susceptible to developing BC [67]. This is in line with a recent study that reported tamoxifen-resistant BC cells display resistance to DNA-damaging therapy due to elevated levels of BRCA1 [68]. Previously, BRCA1 expression has been correlated to a poor prognosis in patients with early BC, which suggests that CRISPR/Cas9 targeting PI3K may be a promising approach to combat chemo-resistance, as PI3K inhibition resulted in impaired BRAC1/2 gene and sensitize BC cells to drugs responses [69].

### CRISPR in identifying mechanism of drug resistance

CRISPR-Cas9-dependent genome-wide screening has also been used to find the drug resistance mechanisms to protein kinase inhibitors in TNBC cell lines. Studies have reported that anaphase-promoting complex (cyclosome)-associated with TTK protein kinase acts as an inhibitor of BC cell lines [70]. In another study using CRISPR/Cas9, the genome-wide assessment to screen cytotoxic T-cells for tumour cell resistance could potentially assist in evaluating the metabolic activities based upon tumour genetics networks [71]. High-throughput analyses of the CRISPR/Cas9-based library are very promising in assessing the genes mutations for treatment failures with high accuracy and precision [72]. Additionally, in cancer immunotherapy, a genome-scale CRISPR/Cas9-based library containing around 123,000 single guide RNAs (sgRNA) have been employed to disrupt genes in melanoma cells to simulate the T cell-based therapy to overcome drug resistance developed due to loss-of-functional mutations [73]. Furthermore, CRISPR/Cas9 has been applied to identify the loss of functional mutation in apelin receptor (APLNR), involved in immunotherapeutic resistance. Since APLNR interacts with JAK1 and modulates IFN-γ responses in cancers, and its deletions can reduce the efficiency of T cell therapy [73].

Furthermore, genome wide CRISPR/Cas9 knockout has been utilized to identify two key regulatory (mTOR and Hippo) pathways involved in tumorigenesis of TNBC under in vivo xenograft model. In this study, Dia et al. highlighted that mTORC2/RICTOR and Sestrin3/GATOR2/WDR59 are very important in enhancing TNBC pathogenesis [74]. Hence, CRISPR/Cas9 could be an important approach in circumventing tumorigenesis and reducing tumor load for BC.

The germline genetic variants also influence the drug metabolic pathways. The report has shown that single nucleotide polymorphisms (SNP) in the zinc finger proteins (ZNF423) gene acts as a predictive indicator for the responsiveness to ER modulator drugs in BC patients [75]. In another study, CRISPR/Cas tool was employed to generate wild-type cells from ERα positive ZR75–1 cells containing the rs9940645 variant, which shows more responsiveness to different drugs such as raloxifene, olaparib, and cisplatin in BC patients [75]. The genetic alterations in T47D and MCF7 cancer cells with ESR1 mutations have resulted in the resistance to fulvestrant, raloxifene, and 4-Hydroxytamoxifen (4-OHT) drugs under in vitro conditions [76]. Hence CRISPR/Cas9 system could play a crucial role in sensitizing the cancer cells to drug response, which could further aid in improving the patient outcomes.

### CRISPR/Cas9 in sensitizing the cells to drugs

The deletion of the MALAT-1 promoter in the BT-549 TNBC model using CRISPR/Cas9 increased susceptibility to paclitaxel and doxorubicin, proposing its role in imparting resistance to the lncRNA transcriptional portrait. Additionally, it also emphasizes a MALAT1-coordinated complex regulatory network for TNBC resistance to neoadjuvant chemotherapy (NAC) [77]. Smad2 and Smad3 protein levels have been shown to be altered in numerous TNBC cell lines in comparison to normal mammary epithelial cells, signifying their possible crucial role in cancer cells escaping TGF-mediated growth inhibition. Thus, the CRISPR/Cas9 system may be very effective for the treatment and prevention of TNBC by selectively targeting the TGF-Smad3-TMEPAI axis [78]. Researchers also reported that a mutant variant of a cellular oncogene could be changed to an inactive form by the CRISPR/Cas9 system. In this regard, CRISPR/Cas9 has been aimed to target the tyrosine kinase domain, which could be utilized to modify the Src family of oncogenes, required for tyrosine kinase activity to transform into an oncogenic form. Also, CRISPR/Cas9 has been utilized in disrupting the functional domains of HER2, needed for its carcinogenic activity, which assists in overcoming treatment resistance [79].

### CRISPR/Cas9 in screening drug resistance gene

Ha et al. utilized CRISPR/Cas9 technology to target multidrug resistance protein 1 (MDR1) in cancer cells in an attempt to eradicate doxorubicin resistance [80]. After disrupting MDR1 using Cas9-sgRNA, doxorubicin was delivered to MCF-7/ADR cells, leading to the recovery of drug sensitivity, which suggests that the drug potency could be enhanced by inducing mutation in drug-resistant genes using CRISPR/Cas9. Furthermore, CRISPR/Cas9 technique has been proposed also for genome-scale deletion and transcriptional activation screening and possesses immense potential for drug-resistant gene screening in a shorter duration [81]. As evidenced by CRISPR/Cas9, the germline mutations in the BRCA1 gene could be overcome through somatic alternative splicing, leading to therapeutic resistance to cancer [82]. It has been reported that TNBC is diagnosed in at least one-third of patients with BRCA1 mutations [83]. TNBC is the most aggressive type of cancer and is tough to cure not only due to the lack of molecular target receptors but also the presence of BRCA1 mutations causing chemotherapeutic drug resistance elevating risks of disease recurrence. However, the synthetic lethal pair of BRCA1, the poly (ADP-ribose) polymerase 1 (PARP1) gene, has been shown as conserved in mostly BRAC1 mutated (BRCA1m) cancer cells and seems to be a promising pathway in sensitizing these cells to chemotherapy. Using CRISPR/Cas9 technology, the generated PARP1-deficient TNBC cell lines, i.e., MDA-MB-231 (BRCA1 wild-type) and MDA-MB-436 (BRCA1m) results in their increased sensitization, leading to reduced dose essential for therapeutic efficacy of the drugs [84]. This also revealed that TNBC cells co-expressing BRCA1m and PARP1m were highly vulnerable to three chemotherapeutic BC drugs such as doxorubicin, gemcitabine, and docetaxel, in a 2D culture environment than their wild-type counterparts [84].

Researchers developed a CRISPR/Cas9-mediated knock-in mutational model to examine the resistance mechanisms of metastatic BC to anti-ER treatments. Because the ER has been identified as the main driving factor for BC progression, and its decreased activity has been demonstrated in reducing the risk of relapse and increasing patient survival [43]. Despite this, cancers can develop resistance to anti-ER therapy while still being ER-positive, cancer cells were sensitive to treatment in many circumstances [85]. Mutations in ER genes, which are uncommon in primary BC, have been associated with drug resistance [86]. Estrogen receptor gene 1 (ESR1) has been revealed as the most common gene in patients who had undergone endocrine therapy for advanced BC [87]. Furthermore, treatment-induced resistance implies that ESR1 mutations develop resistance under treatment-selective stress [88]. To understand this, the CRISPR-Cas9 system was utilized for genome editing to make ESR1 a single allele at mutated amino acid residue, tyrosine 537 in ER-sensitive MCF7- BC cell line [89]. This suggested that ER mutation plays a crucial role in drug resistance during chemotherapy for BCs.

The major multi-specific MAP transporter, which carries the anti-cancer drugs outside to cancer cells has been identified as Ral-interacting protein (RLIP). The abnormal expression of RLIP causes therapeutic resistance in a variety of malignancies. In BC cells transduced with LV vectors containing RLIP sgRNAs, the Cas9 expression damage the RLIP gene, thereby limiting BC cell proliferation under both in vitro and in vivo conditions. RLIP downregulation induces apoptosis via both drug-dependent and independent pathways, and these findings suggest that RLIP may be a viable target for killing cancer cells [90] (Fig. 2).

## Advancement in CRISPR/Cas9

The homology-directed repair mechanism (HDR) in comparison to NHEJ is more specific and is predominantly employed to generate gene knock-in for targeted gene editing [91]. NHEJ is functional during the complete cell cycle, whereas the HDR pathway works only in S and G2/M phases [92], which limits the HDR-driven gene editing in actively dividing cells, restricting its therapeutic potential because stem cells are obtained in a dormant stage [93]. To overcome this, the advanced technique homology-independent targeted integration (HITI) may be utilized to produce gene knock-ins in both dividing and non-dividing cells using NHEJ [16]. HITI is a more efficient technique in producing gene knock-ins than HDR. Nonetheless, these different restrictions should be sorted out before HITI utilize in clinical sett-up. For example, the knock-in capacity of HITI is very low approximately less than 5% in dormant cells [94], and also off-target effects (OTEs) of Cas9 may cause transgene insertion at non-target sites as well. Therefore, using specifically selected Cas9/gRNA target sequences and highly specific Cas9 nuclease, this kind of OTEs with HITI could be minimized [95].

An alternative, end-joining technique MMEJ (microhomology-mediated end-joining) may be also utilized in CRISPR/Cas9 system for genome alteration. MMEJ is activated when there is microhomology (5–25 bp) upstream and downstream of DSBs [96]. This permits two microhomology sequences to be annealed, leading to the elimination of the intervening sequence [96]. Nakade et al. have established an MMEJ-based technique for targeted knock-in of transgenes, named precise integration into target chromosome (PITCh) [96]. Cas9 cleavage of the PITCh donor vector and the genome reveals their microhomology sequences, triggering MMEJ-mediated incorporation of transgenes into the genome at the DSBs [96]. MMEJ is functional while HDR is dormant during the M and early S phase [97], moreover, MMEJ is 2–3 times more efficacious than HDR in accomplishing targeted transgene knock-in [96].

Base editing can facilitate the conversion of the four transition mutations, but not transversion mutations. Anzalone et al. have described a dynamic prime-editing technique that can perform targeted insertions, deletions, and conversions of all 12-pointmutation combinations scans the requirement of a donor template [98]. Prime editing necessitates the use of two elements- Cas9 nickase and a prime editing guide RNA (pegRNA) which is an extended variant of sgRNA including a primer binding site to enable hybridization of the 3' end of the sliced genomic DNA and a reverse transcriptase (RT) template carrying the desired modification to provide a template for the generation of the modified information [98]. The catalytically inhibited Cas9 nickase is linked to an RT and forms a single-strand snip in genomic DNA to enable the 3' end nick and attach to the primer binding site of the pegRNA [98]. Hence, the RT reverse transcribes the sequence data, comprising the edit from the RT template to the DNA [98]. Concerning efficiency, genotoxicity, and adaptability in gene editing, prime editing seems to be stronger than other editing techniques at the moment [98]. Nonetheless, more research into this method in different cell types, as well as refinement of the delivery strategy is required.

## Limitations of CRISPR/Cas9

CRISPR/Cas9 genome editing, which targets coding and non-coding region on chromosomes, has proven to be a valuable method for basic research as well as therapeutic applications in BC. Though, it has been also linked to promoting carcinogenesis. On the other hand, CRISPR/Cas9 causes both genotoxicity and immunotoxicity, in which, genotoxicity has been associated with off-target effects. This technological challenge has delayed the development of CRISPR/Cas9-based cancer therapeutics, since these issues cause mutations that may be carcinogenic to humans [43]. Other challenges have been also associated with the mode of delivery for CRISPR/Cas9 within target cells. Currently, there are two delivery methods i.e., viral and bacteriophage-derived vectors being employed for the CRISPR/Cas9, which may cause genotoxicity and cellular toxicity [99]. To overcome this, the encapsulation of the CRISPR/Cas9 system inside lipopolymer with cell-specific aptamer could be an excellent method for its delivery, which could enable cancer-specific targeting and reduce toxicity as compared to standard viral and non-viral delivery methods [100].

The lack of antigen-specific T-cells focused against the Cas9 protein is one of the major issues in engaging the CRISPR/Cas9 technology for therapeutic medications. Chew recently investigated the potential immunological risks of CRISPR/Cas9 generated medications in clinical trials [101]. Another recent study found that human cells had pre-existing and adaptive immunological responses to bacteria-derived Cas9 proteins [102]. This raises serious concerns regarding the efficacy and, more prominently, the safety of the CRISPR/Cas9 method in treating diseases. Hence, further research is needed to fully understand the role of Cas9-specific T-cells during immunotherapy. These investigations should also look towards creating a Cas9 that can evade the host immune system or at the very least fusion of an immune-compromised molecule into the Cas9-harboring cassette [103].

The CRISPR/Cas9 technology has a few other drawbacks also such as off-target alterations, which may be highly deleterious. Also, CRISPR/Cas9 genome editing in living cells is fraught with dangers due to the lack of specificity. Off-target cleaving processes have been examined comprehensively, and a set of fundamental criteria for limiting off-target effects in research has emerged. For resolving the low-editing efficiency of CRISPR/Cas9 in some specific loci, a “pop-in/pop-out” approach has been designed by Cech et al. for enhancing edited clones that have undergone homologous recombination [104]. This can also be utilized for the screening of effective gene manipulation, particularly for inaccessible loci. To reduce off-target effects, sgRNA should be designed with high precision, particularly at the 5′end sequence [103]. On the other hand, Cas9 activityis regarded as a critical element in identifying the off-target effect. The higher the Cas9 activity, the greater the number of off-targets due to non-specific cleavage. Thus, modifying Cas9 activity could benefit by decreasing off-target effects [105]. On-target is often determined by various notable parameters, such as gRNA design, Cas9 structure, gRNA/Cas9 ratio, and ultimately target site originality [106]. Several techniques have been proposed to address the off-targeting problem, including reducing gRNAs to 20 bases, which might boost specificity by 5000-fold [95]. Switching the electric polarity of Cas9’s two domains, HNH and RuvC, to minimize off-target editing may be more precise with a lower off-target score [107]. The introduction of Cas9 in its protein form instead of as Cas9-plasmid DNA improves on-target performance and diminishes off-target alteration [108].

## Future prospects and conclusion

CRISPR/Cas9 is a ground breaking tool, which has been employed to treat various diseases including cancers. Since, it possesses characteristics of simple genome editing skills in the terms of cost-effectiveness, high specificity, precision, and shorter time duration without the need for multi-functional mice colonies. Hence, it gained a huge interest in the scientific world especially in the field of cancer biology. The use of the CRISPR/Cas9 system raises various social and ethical concerns, not only for human beings but also for other organisms and the environment, such as safety for its use in genetic enhancement [79]. Ethical issues have been raised regarding the prospect of human germline genome editing such that the genetic information can be transmitted down through generations via gametes, first embryo divisions, or fertilized eggs. However, a few concerns need to be addressed such as off-target effects and delivery methods. The CRISPR/Cas9 technology has been adopted by modern researchers primarily for the suppression of oncogenes and activation of TSGs in mouse models. To note, the development of BC is not only ascribed to genetic alterations but also epigenetic changes, which could be tackled through CRISPR methodology.

Moreover, the possibilities of gene therapy using CRISPR/Cas9 remain a promising even though a few technical obstacles exist in targeting cancer genes. CRISPR/Cas9-based techniques will hopefully become a better tactic in the future personalized medicine to deal with the complexities of various tumours and cancer drug resistance. Notwithstanding, the effectiveness of CRISPR/Cas9-mediated therapy will rely on carefully designed sgRNA, monitoring of potential off-target effects, and efficient delivery. From fundamental research to clinical implementation, this technique has opened promising possibilities for the treatment of chemotherapeutic drug resistance.

## Data Availability

Not applicable.

## References

[1] Jagadish N, Gupta N, Agarwal S, Parashar D, Sharma A, Fatima R (2016). Sperm-associated antigen 9 (SPAG9) promotes the survival and tumor growth of triple-negative breast cancer cells. Tumor Biol.

[2] Siegel RL, Miller KD, Fuchs HE, Jemal A (2022). Cancer statistics, 2022. CA Cancer J Clin.

[3] Curigliano G (2012). New drugs for breast cancer subtypes: targeting driver pathways to overcome resistance. Cancer Treat Rev.

[4] Giuliano M, Hu H, Wang YC, Fu X, Nardone A, Herrera S (2015). Upregulation of ER signaling as an adaptive mechanism of cell survival in HER2-positive breast tumors treated with anti-HER2 therapy. Clin Cancer Res.

[5] Minicozzi P, Bella F, Toss A, Giacomin A, Fusco M, Zarcone M (2013). Relative and disease-free survival for breast cancer in relation to subtype: a population-based study. J Cancer Res Clin Oncol.

[6] Toss A, Venturelli M, Peterle C, Piacentini F, Cascinu S, Cortesi L (2017). Molecular biomarkers for prediction of targeted therapy response in metastatic breast cancer: trick or treat?. Int J Mol Sci.

[7] Paterson R, Phillips KA (2017). Genetic testing in women with breast cancer: implications for treatment. Expert Rev Anticancer Ther.

[8] Vogelstein B, Papadopoulos N, Velculescu VE, Zhou S, Diaz LA, Kinzler KW (2013). Cancer genome landscapes. Science.

[9] Havas KM, Milchevskaya V, Radic K, Alladin A, Kafkia E, Garcia M (2017). Metabolic shifts in residual breast cancer drive tumor recurrence. J Clin Invest.

[10] Banerjee B, Sherwood RI (2017). A CRISPR view of gene regulation. Curr Opin Syst Biol.

[11] Chen X, Liu J, Janssen JM, Gonçalves MAFV (2017). The chromatin structure differentially impacts high-specificity CRISPR-Cas9 nuclease strategies. Mol Ther Nucleic Acids.

[12] Fu Y, Sander JD, Reyon D, Cascio VM, Joung JK (2014). Improving CRISPR-Cas nuclease specificity using truncated guide RNAs. Nat Biotechnol.

[13] Dang Y, Jia G, Choi J, Ma H, Anaya E, Ye C (2015). Optimizing sgRNA structure to improve CRISPR-Cas9 knockout efficiency. Genome Biol.

[14] Sun B, Chen H, Gao X (2021). Versatile modification of the CRISPR/Cas9 ribonucleoprotein system to facilitate in vivo application. J Control Release.

[15] Karlson CKS, Mohd-Noor SN, Nolte N, Tan BC (2021). CRISPR/dCas9-based systems: mechanisms and applications in plant sciences. Plants.

[16] Cong L, Zhang F (2015). Genome engineering using CRISPR-Cas9 system. Methods Mol Biol.

[17] Narimani M, Sharifi M, Jalili A (2019). Knockout Of BIRC5 gene By CRISPR/Cas9 induces apoptosis and inhibits cell proliferation in leukemic cell lines, HL60 And KG1. Blood Lymphat Cancer.

[18] Hu Z, Yu L, Zhu D, Ding W, Wang X, Zhang C (2014). Disruption of HPV16-E7 by CRISPR/Cas system induces apoptosis and growth inhibition in HPV16 positive human cervical cancer cells. BioMed Res Int.

[19] Kawamura N, Nimura K, Nagano H, Yamaguchi S, Nonomura N, Kaneda Y (2015). CRISPR/Cas9-mediated gene knockout of NANOG and NANOGP8 decreases the malignant potential of prostate cancer cells. Oncotarget.

[20] Cai J, Huang S, Yi Y, Bao S (2019). Ultrasound microbubble-mediated CRISPR/Cas9 knockout of C-erbB-2 in HEC-1A cells. J Int Med Res.

[21] George J, Li Y, Kadamberi IP, Parashar D, Tsaih S-W, Gupta P (2021). RNA-binding protein FXR1 drives cMYC translation by recruiting eIF4F complex to the translation start site. Cell Rep.

[22] Parashar D, Geethadevi A, Aure MR, Mishra J, George J, Chen C (2019). miRNA551b-3p activates an oncostatin signaling module for the progression of triple-negative breast cancer. Cell Rep.

[23] Schuijers J, Manteiga JC, Weintraub AS, Day DS, Zamudio AV, Hnisz D (2018). Transcriptional dysregulation of MYC reveals common enhancer-docking mechanism. Cell Rep.

[24] Chen D, Zhao Z, Huang Z, Chen D-C, Zhu X-X, Wang Y-Z (2018). Super enhancer inhibitors suppress MYC driven transcriptional amplification and tumor progression in osteosarcoma. Bone Res.

[25] Yoshida M, Yokota E, Sakuma T, Yamatsuji T, Takigawa N, Ushijima T (2018). Development of an integrated CRISPRi targeting ΔNp63 for treatment of squamous cell carcinoma. Oncotarget.

[26] Luo S, Chen J, Mo X (2016). The association of PTEN hypermethylation and breast cancer: a meta-analysis. Onco Targets Ther.

[27] Choudhury SR, Cui Y, Lubecka K, Stefanska B, Irudayaraj J (2016). CRISPR-dCas9 mediated TET1 targeting for selective DNA demethylation at BRCA1 promoter. Oncotarget.

[28] Lu A, Wang J, Sun W, Huang W, Cai Z, Zhao G (2019). Reprogrammable CRISPR/dCas9-based recruitment of DNMT1 for site-specific DNA demethylation and gene regulation. Cell Discov.

[29] Yang M, Zeng C, Li P, Qian L, Ding B, Huang L (2019). Impact of CXCR4 and CXCR7 knockout by CRISPR/Cas9 on the function of triple-negative breast cancer cells. Onco Targets Ther.

[30] Wu W, Qian L, Chen X, Ding B (2015). Prognostic significance of CXCL12, CXCR4, and CXCR7 in patients with breast cancer. Int J Clin Exp Pathol.

[31] Burns JM, Summers BC, Wang Y, Melikian A, Berahovich R, Miao Z (2006). A novel chemokine receptor for SDF-1 and I-TAC involved in cell survival, cell adhesion, and tumor development. J Exp Med.

[32] Hannafon BN, Cai A, Calloway CL, Xu Y-F, Zhang R, Fung K-M (2019). miR-23b and miR-27b are oncogenic microRNAs in breast cancer: evidence from a CRISPR/Cas9 deletion study. BMC Cancer.

[33] Curtis C, Shah SP, Chin S-F, Turashvili G, Rueda OM, Dunning MJ (2012). The genomic and transcriptomic architecture of 2,000 breast tumours reveals novel subgroups. Nature.

[34] Gharbi-Ayachi A, Labbé J-C, Burgess A, Vigneron S, Strub J-M, Brioudes E (2010). The substrate of Greatwall kinase, Arpp19, controls mitosis by inhibiting protein phosphatase 2A. Science.

[35] Wurzenberger C, Gerlich DW (2011). Phosphatases: providing safe passage through mitotic exit. Nat Rev Mol Cell Biol.

[36] Álvarez-Fernández M, Sánchez-Martínez R, Sanz-Castillo B, Gan PP, Sanz-Flores M, Trakala M (2013). Greatwall is essential to prevent mitotic collapse after nuclear envelope breakdown in mammals. Proc Natl Acad Sci U S A.

[37] Álvarez-Fernández M, Sanz-Flores M, Sanz-Castillo B, Salazar-Roa M, Partida D, Zapatero-Solana E (2017). Therapeutic relevance of the PP2A-B55 inhibitory kinase MASTL/Greatwall in breast cancer. Cell Death Differ.

[38] Hardwicke MA, Rendina AR, Williams SP, Moore ML, Wang L, Krueger JA (2014). A human fatty acid synthase inhibitor binds β-ketoacyl reductase in the keto-substrate site. Nat Chem Biol.

[39] Gonzalez-Salinas F, Rojo R, Martinez-Amador C, Herrera-Gamboa J, Trevino V (2020). Transcriptomic and cellular analyses of CRISPR/Cas9-mediated edition of FASN show inhibition of aggressive characteristics in breast cancer cells. Biochem Biophys Res Commun.

[40] Wang H, Sun W (2017). CRISPR-mediated targeting of HER2 inhibits cell proliferation through a dominant negative mutation. Cancer Lett.

[41] Bernardo GM, Keri RA (2011). FOXA1: a transcription factor with parallel functions in development and cancer. Biosci Rep.

[42] Zhang G, Zhao Y, Liu Y, Kao L-P, Wang X, Skerry B (2016). FOXA1 defines cancer cell specificity. Sci Adv.

[43] Yang H, Jaeger M, Walker A, Wei D, Leiker K, Weitao T (2018). Break breast cancer addiction by CRISPR/Cas9 genome editing. J Cancer.

[44] Wang Y, Zhang T, Kwiatkowski N, Abraham BJ, Lee TI, Xie S (2015). CDK7-dependent transcriptional addiction in triple-negative breast cancer. Cell.

[45] Sun B, Mason S, Wilson RC, Hazard SE, Wang Y, Fang R (2020). Inhibition of the transcriptional kinase CDK7 overcomes therapeutic resistance in HER2-positive breast cancers. Oncogene.

[46] Bolt MJ, Stossi F, Callison AM, Mancini MG, Dandekar R, Mancini MA (2015). Systems level-based RNAi screening by high content analysis identifies UBR5 as a regulator of estrogen receptor-α protein levels and activity. Oncogene.

[47] Liao L, Song M, Li X, Tang L, Zhang T, Zhang L (2017). E3 ubiquitin ligase UBR5 drives the growth and metastasis of triple-negative breast cancer. Cancer Res.

[48] Zuo T, Wang L, Morrison C, Chang X, Zhang H, Li W (2007). FOXP3 is an X-linked breast cancer suppressor gene and an important repressor of the HER-2/ErbB2 oncogene. Cell.

[49] Cui X, Zhang C, Xu Z, Wang S, Li X, Stringer-Reasor E (2022). Dual CRISPR interference and activation for targeted reactivation of X-linked endogenous FOXP3 in human breast cancer cells. Mol Cancer.

[50] Dekkers JF, Whittle JR, Vaillant F, Chen H-R, Dawson C, Liu K (2020). Modeling breast cancer using CRISPR-Cas9-mediated engineering of human breast organoids. J Natl Cancer Inst.

[51] Azangou-Khyavy M, Ghasemi M, Khanali J, Boroomand-Saboor M, Jamalkhah M, Soleimani M (2020). CRISPR/Cas: from tumor gene editing to T cell-based immunotherapy of cancer. Front Immunol.

[52] Grenier JM, Yeung ST, Khanna KM (2018). Combination immunotherapy: taking cancer vaccines to the next level. Front Immunol.

[53] Eyquem J, Mansilla-Soto J, Giavridis T, van der Stegen SJC, Hamieh M, Cunanan KM (2017). Targeting a CAR to the TRAC locus with CRISPR/Cas9 enhances tumour rejection. Nature.

[54] Russ A, Hua AB, Montfort WR, Rahman B, Riaz IB, Khalid MU (2018). Blocking “don’t eat me” signal of CD47-SIRPα in hematological malignancies, an in-depth review. Blood Rev.

[55] Fan P, He ZY, Xu T, Phan K, Chen GG, Wei Y-Q. Exposing cancer with CRISPR-Cas9: from genetic identification to clinical therapy. Transl Cancer Res. AME Publishing Company; vol. 7; 2018. https://tcr.amegroups.com/article/view/22195. Accessed 9 Oct 2021.

[56] Gurusamy D, Henning AN, Yamamoto TN, Yu Z, Zacharakis N, Krishna S (2020). Multi-phenotype CRISPR-Cas9 screen identifies p38 kinase as a target for adoptive immunotherapies. Cancer Cell.

[57] Deng H, Tan S, Gao X, Zou C, Xu C, Tu K (2020). Cdk5 knocking out mediated by CRISPR-Cas9 genome editing for PD-L1 attenuation and enhanced antitumor immunity. Acta Pharm Sin B.

[58] Chao MP, Weissman IL, Majeti R (2012). The CD47–SIRPα pathway in cancer immune evasion and potential therapeutic implications. Curr Opin Immunol.

[59] Wang G, Chow RD, Bai Z, Zhu L, Errami Y, Dai X (2019). Multiplexed activation of endogenous genes by CRISPRa elicits potent antitumor immunity. Nat Immunol.

[60] Zheng YZ, Xue MZ, Shen HJ, Li XG, Ma D, Gong Y (2018). PHF5A epigenetically inhibits apoptosis to promote breast cancer progression. Cancer Res.

[61] Goldhirsch A, Winer EP, Coates AS, Gelber RD, Piccart-Gebhart M, Thürlimann B (2013). Personalizing the treatment of women with early breast cancer: highlights of the St Gallen International Expert Consensus on the Primary Therapy of Early Breast Cancer 2013. Ann Oncol.

[62] Martinez-Lage M, Puig-Serra P, Menendez P, Torres-Ruiz R, Rodriguez-Perales S (2018). CRISPR/Cas9 for cancer therapy: hopes and challenges. Biomedicines..

[63] Lino CA, Harper JC, Carney JP, Timlin JA (2018). Delivering CRISPR: a review of the challenges and approaches. Drug Deliv.

[64] Bleau A-M, Hambardzumyan D, Ozawa T, Fomchenko EI, Huse JT, Brennan CW (2009). PTEN/PI3K/Akt pathway regulates the side population phenotype and ABCG2 activity in glioma tumor stem-like cells. Cell Stem Cell.

[65] Jung SH, Hwang HJ, Kang D, Park HA, Lee HC, Jeong D (2019). mTOR kinase leads to PTEN-loss-induced cellular senescence by phosphorylating p53. Oncogene.

[66] Phuah S-Y, Looi L-M, Hassan N, Rhodes A, Dean S, Taib NAM (2012). Triple-negative breast cancer and PTEN (phosphatase and tensin homologue)loss are predictors of BRCA1 germline mutations in women with early-onset and familial breast cancer, but not in women with isolated late-onset breast cancer. Breast Cancer Res.

[67] Moses C, Nugent F, Waryah CB, Garcia-Bloj B, Harvey AR, Blancafort P (2019). Activating PTEN tumor suppressor expression with the CRISPR/dCas9 system. Mol Ther Nucleic Acids.

[68] Zhu Y, Liu Y, Zhang C, Chu J, Wu Y, Li Y (2018). Tamoxifen-resistant breast cancer cells are resistant to DNA-damaging chemotherapy because of upregulated BARD1 and BRCA1. Nat Commun.

[69] Biagioni A, Laurenzana A, Margheri F, Chillà A, Fibbi G, Del Rosso M (2018). Delivery systems of CRISPR/Cas9-based cancer gene therapy. J Biol Eng.

[70] King JL, Zhang B, Li Y, Li KP, Ni JJ, Saavedra HI (2018). TTK promotes mesenchymal signaling via multiple mechanisms in triple negative breast cancer. Oncogenesis.

[71] Zhao D, Badur MG, Luebeck J, Magaña JH, Birmingham A, Sasik R (2018). Combinatorial CRISPR-Cas9 metabolic screens reveal critical redox control points dependent on the KEAP1-NRF2 regulatory axis. Mol Cell.

[72] Shalem O, Sanjana NE, Zhang F (2015). High-throughput functional genomics using CRISPR–Cas9. Nat Rev Genet.

[73] Patel SJ, Sanjana NE, Kishton RJ, Eidizadeh A, Vodnala SK, Cam M (2017). Identification of essential genes for cancer immunotherapy. Nature.

[74] Dai M, Yan G, Wang N, Daliah G, Edick AM, Poulet S (2021). In vivo genome-wide CRISPR screen reveals breast cancer vulnerabilities and synergistic mTOR/Hippo targeted combination therapy. Nat Commun.

[75] Qin S, Ingle JN, Liu M, Yu J, Wickerham DL, Kubo M (2017). Calmodulin-like protein 3 is an estrogen receptor alpha coregulator for gene expression and drug response in a SNP, estrogen, and SERM-dependent fashion. Breast Cancer Res.

[76] Bahreini A, Li Z, Wang P, Levine KM, Tasdemir N, Cao L (2017). Mutation site and context dependent effects of ESR1 mutation in genome-edited breast cancer cell models. Breast Cancer Res.

[77] Shaath H, Vishnubalaji R, Elango R, Khattak S, Alajez NM (2021). Single-cell long noncoding RNA (lncRNA) transcriptome implicates MALAT1 in triple-negative breast cancer (TNBC) resistance to neoadjuvant chemotherapy. Cell Death Discov.

[78] Singha PK, Pandeswara S, Geng H, Lan R, Venkatachalam MA, Dobi A (2019). Increased Smad3 and reduced Smad2 levels mediate the functional switch of TGF-β from growth suppressor to growth and metastasis promoter through TMEPAI/PMEPA1 in triple negative breast cancer. Genes Cancer.

[79] Chen Y, Zhang Y (2018). Application of the CRISPR/Cas9 system to drug resistance in breast cancer. Adv Sci.

[80] Ha JS, Byun J, Ahn D-R (2016). Overcoming doxorubicin resistance of cancer cells by Cas9-mediated gene disruption. Sci Rep.

[81] Joung J, Konermann S, Gootenberg JS, Abudayyeh OO, Platt RJ, Brigham MD (2017). Genome-scale CRISPR-Cas9 knockout and transcriptional activation screening. Nat Protoc.

[82] Wang Y, Bernhardy AJ, Cruz C, Krais JJ, Nacson J, Nicolas E (2016). The BRCA1-Δ11q alternative splice isoform bypasses germline mutations and promotes therapeutic resistance to PARP inhibition and cisplatin. Cancer Res.

[83] Singh DD, Han I, Choi E-H, Yadav DK (2021). CRISPR/Cas9 based genome editing for targeted transcriptional control in triple-negative breast cancer. Comput Struct Biotechnol J.

[84] Mintz RL, Lao Y-H, Chi C-W, He S, Li M, Quek CH (2020). CRISPR/Cas9-mediated mutagenesis to validate the synergy between PARP1 inhibition and chemotherapy in BRCA1-mutated breast cancer cells. Bioeng Transl Med.

[85] Ali S, Buluwela L, Coombes RC (2011). Antiestrogens and their therapeutic applications in breast cancer and other diseases. Annu Rev Med.

[86] Segal CV, Dowsett M (2014). Estrogen receptor mutations in breast cancer—new focus on an old target. Clin Cancer Res.

[87] Jeselsohn R, Yelensky R, Buchwalter G, Frampton G, Meric-Bernstam F, Gonzalez-Angulo AM (2014). Emergence of constitutively active estrogen receptor-α mutations in pretreated advanced estrogen receptor-positive breast cancer. Clin Cancer Res.

[88] Guttery DS, Page K, Hills A, Woodley L, Marchese SD, Rghebi B (2015). Noninvasive detection of activating estrogen receptor 1 (ESR1) mutations in estrogen receptor-positive metastatic breast cancer. Clin Chem.

[89] Harrod A, Fulton J, Nguyen VTM, Periyasamy M, Ramos-Garcia L, Lai C-F (2017). Genomic modelling of the ESR1 Y537S mutation for evaluating function and new therapeutic approaches for metastatic breast cancer. Oncogene.

[90] Singhal J, Chikara S, Horne D, Awasthi S, Salgia R, Singhal SS (2021). Targeting RLIP with CRISPR/Cas9 controls tumor growth. Carcinogenesis.

[91] Hsu PD, Scott DA, Weinstein JA, Ran FA, Konermann S, Agarwala V (2013). DNA targeting specificity of RNA-guided Cas9 nucleases. Nat Biotechnol.

[92] Ranjha L, Howard SM, Cejka P (2018). Main steps in DNA double-strand break repair: an introduction to homologous recombination and related processes. Chromosoma.

[93] Li L, Bhatia R (2011). Stem cell quiescence. Clin Cancer Res.

[94] Suzuki K, Izpisua Belmonte JC (2018). In vivo genome editing via the HITI method as a tool for gene therapy. J Hum Genet.

[95] Kleinstiver BP, Pattanayak V, Prew MS, Tsai SQ, Nguyen NT, Zheng Z (2016). High-fidelity CRISPR–Cas9 nucleases with no detectable genome-wide off-target effects. Nature.

[96] Nakade S, Tsubota T, Sakane Y, Kume S, Sakamoto N, Obara M (2014). Microhomology-mediated end-joining-dependent integration of donor DNA in cells and animals using TALENs and CRISPR/Cas9. Nat Commun.

[97] Sakuma T, Nakade S, Sakane Y, Suzuki K-IT, Yamamoto T (2016). MMEJ-assisted gene knock-in using TALENs and CRISPR-Cas9 with the PITCh systems. Nat Protoc.

[98] Anzalone AV, Randolph PB, Davis JR, Sousa AA, Koblan LW, Levy JM (2019). Search-and-replace genome editing without double-strand breaks or donor DNA. Nature.

[99] Yang Y, Wang L, Bell P, McMenamin D, He Z, White J (2016). A dual AAV system enables the Cas9-mediated correction of a metabolic liver disease in newborn mice. Nat Biotechnol.

[100] Liang C, Li F, Wang L, Zhang Z-K, Wang C, He B (2017). Tumor cell-targeted delivery of CRISPR/Cas9 by aptamer-functionalized lipopolymer for therapeutic genome editing of VEGFA in osteosarcoma. Biomaterials.

[101] Chew WL (2018). Immunity to CRISPR Cas9 and Cas12a therapeutics. WIREs Syst Biol Med.

[102] Charlesworth CT, Deshpande PS, Dever DP, Camarena J, Lemgart VT, Cromer MK (2019). Identification of preexisting adaptive immunity to Cas9 proteins in humans. Nat Med.

[103] Sabit H, Abdel-Ghany S, Tombuloglu H, Cevik E, Alqosaibi A, Almulhim F (2021). New insights on CRISPR/Cas9-based therapy for breast Cancer. Genes Environ.

[104] Xi L, Schmidt JC, Zaug AJ, Ascarrunz DR, Cech TR (2015). A novel two-step genome editing strategy with CRISPR-Cas9 provides new insights into telomerase action and TERT gene expression. Genome Biol.

[105] Gong S, Yu HH, Johnson KA, Taylor DW (2018). DNA unwinding is the primary determinant of CRISPR-Cas9 activity. Cell Rep.

[106] Klose RJ, Cooper S, Farcas AM, Blackledge NP, Brockdorff N (2013). Chromatin sampling—an emerging perspective on targeting polycomb repressor proteins. PLoS Genet.

[107] Kimberland ML, Hou W, Alfonso-Pecchio A, Wilson S, Rao Y, Zhang S (2018). Strategies for controlling CRISPR/Cas9 off-target effects and biological variations in mammalian genome editing experiments. J Biotechnol.

[108] Zhang XH, Tee LY, Wang XG, Huang QS, Yang SH. Off-target Effects in CRISPR/Cas9-mediated Genome Engineering. Mol Ther Nucleic Acids. Elsevier; Vol. 4; 2015. https://www.cell.com/molecular-therapy-family/nucleic-acids/abstract/S2162-2531(16)30049-X. Accessed 2 Dec 2021. 10.1038/mtna.2015.37 PMC4877446 26575098

